# How the Cigarette Fire Burns the Rheumatoid Joints

**DOI:** 10.31662/jmaj.2025-0361

**Published:** 2025-09-19

**Authors:** Iris Yan Ki Tang, Chi Chiu Mok

**Affiliations:** 1 Division of Rheumatology & Clinical Immunology, Department of Medicine, The University of Hong Kong, Hong Kong; 2 Department of Medicine, Tuen Mun Hospital, Hong Kong

**Keywords:** Rheumatoid Arthritis, Smoking, Citrullination

Smoking is a preventable environmental risk factor for developing rheumatoid arthritis (RA), especially in genetically susceptible individuals. Beyond increasing RA incidence, smoking also exacerbates disease activity, promotes extra-articular manifestations, and increases cardiovascular risks in RA. A meta-analysis by Di Giuseppe et al. ^(1)^ revealed a 26% increased risk of RA even at low lifetime exposure (≤10 pack-years), with risk plateauing at a 200% increase for exposures exceeding 20 pack-years. While the precise mechanisms linking smoking to the development and progression of RA remain elusive, the review article by Kayo Masuko highlights the emerging evidence on the potential pathogenic roles of smoking in triggering autoimmunity and disrupting gut microbiota ^(2)^.

In the article, Kayo Masuko explores how smoking-induced post-translational protein modifications, particularly citrullination, contribute to RA pathogenesis. Cigarette smoking upregulates Fas (CD95) expression on CD4+ T and B lymphocytes, increasing their vulnerability to apoptotic cell death. Similarly, tobacco smoke induces apoptosis in airway epithelial cells, releasing intracellular citrullinated antigens. This may breach immune tolerance and drive autoantibody production. The presence of anti-citrullinated protein antibodies (ACPAs), a hallmark feature of RA, often precedes clinical disease onset for years. Citrullination, which refers to the conversion from arginine to citrulline, is catalyzed by peptidyl-arginase deiminases (PADs). Smoking has been shown to increase the PAD expression in the lungs, leading to the formation of citrullinated peptides formation and ACPA production. Citrullination also occurs in extrapulmonary tissues, including inflamed synovial tissues. Nicotine in cigarettes further exacerbates RA pathogenesis by inducing the formation of neutrophil extracellular traps (NETs), which are networks of extracellular fibers composed of DNA and proteins released from neutrophils. Corsiero et al. ^(3)^ reviewed the role of neutrophils in RA and reported that RA neutrophils exhibit higher spontaneous NETosis, releasing active PAD (particularly PAD4) into the synovial fluid, which citrullinate histones and perpetuates autoimmunity. The effect of cigarettes on other post-translation protein modifications was also discussed in the article by Kayo Masuko, though their exact pathogenic roles in RA remain unclear.

While the mechanism of smoking-induced citrullination is entailed in the review, it is critical to recognize this effect is largely confined to ACPA-positive RA. The possible interaction between citrullination, smoking and genetic susceptibility was further elaborated in a review article by Klareskog et al. ^(4)^ The evolution of ACPA-positive RA has been hypothesized to begin with local activation of PAD following exposure to cigarette smoke, generating citrullinated proteins/peptides in the lungs. Toxic components in cigarette smoke can activate antigen-presenting cells, increasing the presentation of citrullinated peptides in genetically predisposed individuals, such as those carrying *HLA-DRB1* shared epitope alleles or Protein Tyrosine Phosphatase Non-Receptor Type 22 variants. Antigen-presenting cells activate CD4+ T cells, triggering B-cell mediated ACPA production. This association with major histocompatibility complex class II risk variants is markedly weakened in ACPA-negative RA. A second inflammatory hit (e.g., infection or trauma) subsequently drives the progression from preclinical to clinical RA. Protein citrullination in the synovium and the recruitment of ACPAs to the joint then lead to the formation of immune complexes, release of proinflammatory cytokines, and further activation of pathogenic T and B cells, resulting in joint inflammation and destruction. Over time, the immune response to citrullinated epitopes evolves from a restricted ACPA repertoire in the preclinical stage to recognition of diverse citrullinated autoantigens and exogenous antigens in established RA ^(3)^.

The interaction between smoking and other environmental risk factors in RA has been an immense area of interest. In this review by Kayo Masuko, smoking-related distortion in microbiota and its role in RA pathogenesis is summarized. Nicotine in cigarettes impairs gingival blood flow and weakens immunity in the oral cavity, elevating the risk of gingivitis and periodontitis. *Porphyromonas gingivalis*, a periodontal pathogen, can produce PAD and citrullinated peptides in the oral cavity, triggering subsequent mucosal T cell response. Smoking also causes the dysbiosis in the gut microbiome, compromising the gut barrier integrity and facilitating bacterial translocation. Molecular mimicry between microbial and self-antigens may contribute to autoimmunity in RA.

In addition to increasing the risk of RA onset, cigarette smoking also negatively impacts treatment response. In a Swedish study comparing treatment responses in more than 900 RA patients initiating their first anti-tumor necrosis factor (anti-TNF) treatment ^(5)^, current smoking was a negative predictor for treatment response for up to 12 months. Current smokers were observed to start their first anti-TNF drug sooner than never-smokers. Heavy smokers with >20 pack-years had the worst drug survival, but whether smoking cessation could lead to improved treatment responses remains certain. This underscores the importance of advocate against smoking initiation in general public, particularly among the youth.

Apart from cigarette smoking as discussed in the review, the emerging use of e-cigarettes may represent a novel environmental risk factor for RA. E-cigarettes deliver nicotine and other chemicals such as propylene glycol via aerosol. The use of e-cigarettes is gaining popularity, especially among teenagers and young adults, and is often marketed as a safer alternative to traditional cigarette. However, there is rising concern about whether vaping mirrors traditional smoking in perpetuating gene-environment interactions that ignite autoimmunity. Future studies comparing RA incidence in vapers and traditional cigarette smokers are essential to clarify the risk.

Ultimately, the intricate relationship between smoking and RA pathogenesis offers important insights into the interplay of environmental triggers, genetic susceptibility, and immune dysregulation. By unravelling the role of smoking in RA, researchers can advance the understanding of gene-environment interactions, which might enable identification of biomarkers for early RA or novel therapeutic strategies.

## Article Information

### Conflicts of Interest

None

### Disclaimer

Chi Chiu Mok is one of the Editors of JMA Journal and on the journal’s Editorial Staff. He was not involved in the editorial evaluation or decision to accept this article for publication at all.
